# The Impact of the Immune System on Tumor: Angiogenesis and Vascular Remodeling

**DOI:** 10.3389/fonc.2014.00069

**Published:** 2014-04-08

**Authors:** Christian Stockmann, Dirk Schadendorf, Ralph Klose, Iris Helfrich

**Affiliations:** ^1^UMR 970, Paris Cardiovascular Research Center, Institut National de la Santé et de la Recherche Médicale (INSERM), Paris, France; ^2^Skin Cancer Unit, Dermatology Department, Medical Faculty, University Duisburg-Essen, Essen, Germany

**Keywords:** microenvironment, immune cells, leukocytes, endothelial cells, angiogenesis

## Abstract

Angiogenesis, the formation of new blood vessels, as well as inflammation with massive infiltration of leukocytes are hallmarks of various tumor entities. Various epidemiological, clinical, and experimental studies have not only demonstrated a link between chronic inflammation and cancer onset but also shown that immune cells from the bone marrow such as tumor-infiltrating macrophages significantly influence tumor progression. Tumor angiogenesis is critical for tumor development as tumors have to establish a blood supply in order to progress. Although tumor cells were first believed to fuel tumor angiogenesis, numerous studies have shown that the tumor microenvironment and infiltrating immune cell subsets are important for regulating the process of tumor angiogenesis. These infiltrates involve the adaptive immune system including several types of lymphocytes as well as cells of the innate immunity such as macrophages, neutrophils, eosinophils, mast cells, dendritic cells, and natural killer cells. Besides their known immune function, these cells are now recognized for their crucial role in regulating the formation and the remodeling of blood vessels in the tumor. In this review, we will discuss for each cell type the mechanisms that regulate the vascular phenotype and its impact on tumor growth and metastasis.

## Introduction

Angiogenesis, which is the outgrowth of new vessels from pre-existing capillaries and post-capillary venules, is crucial embryonic development (1). In adults, angiogenesis occurs physiologically in the uterus during the menstrual cycle as well as in pathological conditions, such as the growth of malignant tumors.

In 1971, Folkman generated the hypothesis that tumor growth depends on the neoformation of blood vessels and, thus, inhibition of angiogenesis could prevent tumor progression (2). This work also defined the concept of “anti-angiogenesis” as the prevention of blood vessel recruitment to the tumor. The prediction was that tumors would not grow beyond a minimal size of 1–2 mm^3^ without perfusion and connection to the newly formed capillary network. Consistently, the majority of pre-clinical studies have shown effective inhibition of tumor growth by targeting angiogenic factors. However, the clinical outcome of anti-angiogenic treatment is rather modest as anti-angiogenic drugs improve survival by only a few months (3).

The net angiogenic activity depends on the balance of positive and negative modulators (4) that tightly coordinate the action of various molecules, including, extracellular matrix-degrading enzymes, cellular junction proteins, and cell adhesion receptors, which results in a migratory an invasive behavior of the angiogenic tumor endothelium. In healthy tissue though, the vasculature remains quiescent due to the dominance of negative regulators of angiogenesis (5). Hence, tumor angiogenesis depends on down-regulation of negative regulators as well as a shift toward positive regulators, which are mainly released by neoplastic cells and inflammatory cells that will ultimately lead to the growth of blood vessels (6).

In addition to their increase in density, tumor blood vessels are characterized by various structural and functional abnormalities including irregularities in size and shape, the absence of the typical vessel hierarchy or the distinct organization in arterioles, capillaries, and venules (7). Furthermore, they often exhibit a decreased mural cell coverage and/or abnormal basement membrane sleeves. The endothelial cells that constitute the vascular bed of tumors show a dramatically increased proliferation rate compared to normal endothelial cells resulting in a structurally aberrant and functionally defective vasculature. This distinct vascular phenotype is usually associated with increased permeability that allows the traffic of tumor cells into the circulation (8).

The process of angiogenesis involves a cascade of events including endothelial cell sprouting, the loss of mural cell-endothelial cell association as well as increased vessel permeability (8–10), and the value of vascular density to determine anti-angiogenic activity has been shown to be of limited use (11). Therefore, changes in the functionality of the vasculature are likely to be a more important readout of anti-angiogenic activity than just the presence of a vasculature (8). In fact, recent studies have shown that tumor blood flow and growth are decreased, whereas vessel count is increased (12–15), which further supports the notion that vascular function is more important than simple vessel counts. Indeed in most tumors, despite high vascular density, the blood supply is rather inefficient. Due to the fact that many features of the aberrant tumor vasculature are attributable to the abundance of angiogenic factors like Vascular Endothelial Growth Factor (VEGF), Jain and colleagues have hypothesized that anti-angiogenic therapy can temporarily “normalize” the vascular bed of tumors during the so called window of normalization. The definition of “vascular normalization” includes the reversion of vascular abnormalities (that are, increased permeability, tortuosity, and loss of pericytes) and redistribution of the blood flow with increased delivery of cytotoxic agents and oxygen during the normalization window (8). In fact, in a phase II study with glioblastoma patients, a VEGF receptor tyrosine kinase-inhibitor led to structural and functional to normalization of the tumor vasculature, as measured by MRI (16).

Recent studies gave insight into the causal role of host-derived soluble factors as well as tumor-associated host cells, for initiation and/or progression of cancer (17–20). Recent studies identified the paradox that some leukocytes have the potential to promote, rather than restrict, tumor growth (21, 22). Histological observations of multiple solid tumors revealed the presence of leukocytes within developing tumors as an attempt to eliminate transformed cells. Growing number of reports have implicated tumor-infiltrating immune cells as crucial mediators of cancer initiation and progression (17–19, 23). In addition, type and density of intra-tumoral immune cells have been validated as a reliable parameter for patient’s clinical outcome in certain types of cancer (24–26).

Leukocytes comprise diverse subsets of immune cells that can be separated into cells of the innate and adaptive immunity. The innate immune system consists of macrophages, granulocytes, mast cells, natural killer (NK) cells, and dendritic cells (DCs). Tissue-resident macrophages and mast cells recruit of additional leukocytes from the circulation into the inflamed tissue in response perturbed tissue homeostasis by secreting soluble cytokines and chemokines. Furthermore, DCs have the potential to cross-present antigens to adaptive immune cells, e.g., CD4^+^ T cells and B cells, which in turn undergo clonal expansion resulting in an adaptive immune response against the presented antigen (21).

However, an efficient immune response also depends on the appropriate distribution and positioning of immune cells within dynamic tissue microenvironments. This process is largely controlled by the vascular network and its interactions with circulating immune cells, particularly during pathological circumstances such as inflammation (27, 28). In consequence, vasculature modulated by inflammatory triggers, displays increased leakiness and enhanced leukocyte adhesiveness, resulting in endothelial cell activation, proliferation, and vascular sprouting (29, 30). Thus, there is a well-orchestrated interaction between inflammatory infiltrates and the endothelium. Recent reports further dissected the impact of different immune subsets for blood vessel neoformation and remodeling (20, 23, 31). They functionally contribute to tumor growth and progression by releasing pro-tumorigenic factors like cytokines and chemokines, extracellular matrix-degrading enzymes, reactive oxygen species, and other bioactive molecules, along with angiogenesis and tissue remodeling (20). The appreciation that immune cell-secreted factors might contribute to tumor angiogenesis and in consequence, potentially affect efficacy of anti-angiogenic therapy, identified these cells as a valuable target for anti-cancer strategies (18, 20, 32, 33). To illustrate the different forms of immune cell-EC communication, we will focus on each cell type of the innate and adaptive immunity and their implication on angiogenesis and vascular remodeling.

## Innate Immunity

### Macrophages

Macrophages are specialized phagocytes that are able to incorporate invading microbes and cell debris as well as to secrete release various immunomodulatory cytokines. They have a unique ability to adapt their phenotype to dynamically changing microenvironments that they encounter. The conventional phenotyping distinguishes M1 (classically activated) or M2 (alternatively activated) macrophages. The M1 phenotype is characterized as pro-inflammatory and is associated with T-helper-1 response and the secretion of bactericidal factors in response lipopolysaccharide and interferon γ (IFNγ) exposure. M2 macrophages exhibit a T-helper-2 cytokine expression pattern and are considered to be rather immunosuppressive (34).

The potential role of tumor-associated macrophages (TAMs) in modulating tumor angiogenesis was already proposed in the early 90s (35). After that, a variety of studies have shown that TAMs are often found in the surrounding of blood vessels of solid tumors (36–38). In addition, studies in human tumors demonstrate a positive correlation between blood vessel density and the number of TAMs in vessel areas (39, 40). The pro-angiogenic function of TAMs was also thoroughly investigated in animal cancer models. Accumulating evidences show that TAM depletion results in the decrease of tumor angiogenesis (31, 41), while TAM enhancement exhibits the opposing effect (42). For example, it has been shown that genetic depletion of macrophages in PyMT mammary tumor model delays the angiogenic switch, whereas restoring macrophage infiltration rescues the vessel phenotype (31).

In addition to the functional studies mentioned above, much interest has been given to the mechanistic insights on the pro-angiogenic function of TAMs. Hypoxia occurs quite frequently solid tumors, and macrophages are often attracted to the hypoxic areas of tumor site due to the secretion of hypoxia-induced chemoattractants by tumor cells. Such chemoattractants include VEGF, endothelin, endothelial monocyte activating polypeptide II (EMAP II) (43), and CCL2 (6). Once TAMs are attracted to the hypoxic areas, this microenvironment promotes the metabolic adaptation of TAMs to hypoxia by upregulating hypoxia-inducible factors (HIF)-1, HIF-2, and VEGF (44–46). VEGF-A functions as a potent mitogen for endothelial cells by binding to VEGFR1 and VEGFR2 (47). Genetic studies showed that TAM-derived VEGF-A is essential for angiogenesis in the PyMT mammary tumors (48). Restoring VEGF-A expression in macrophage-deficient PyMT tumor model induces the increase of tumor angiogenesis (48). These data indicate that VEGF is a key regulator of the pro-angiogenic activity of TAMs.

Interestingly, a study using an *in vivo* myeloid cell-specific deletion of VEGF and tested its impact on vessel density and tumor progression in various murine tumor models in order to determine the role of myeloid cell-derived VEGF in this context (49). In the MMTV-PyMT model of mammary tumorigenesis increased vascular density was found as tumors progressed to malignancy, consistent with an “angiogenic switch.” However, in mutant mice with a deletion of VEGF restricted to myeloid cells, the malignancy-associated increase in vascularization, thus the “angiogenic switch,” did not occur. Along with impaired angiogenesis a decrease in vessel length and reduced vessel tortuosity was observed in the absence of myeloid cell-derived VEGF. Although, VEGF protein levels did not vary in tumor lysates from wild type and mutant animals, loss of myeloid-derived VEGF caused an approximately 50% reduction in VEGFR2 phosphorylation, suggesting that myeloid cell-derived VEGF plays an unique role in tumor vascularization, that cannot be compensated for by VEGF from other sources within the tumor. Noteworthy, the onset of tumor growth was not affected by the lack VEGF in myeloid cells. However, surprisingly mutant mice had a significantly higher tumor burden at endpoint than their wild type littermates and along with this a higher number of proliferating cells, indicating that tumors develop at a more rapid pace in the absence of myeloid cell-derived VEGF. Furthermore, the loss of VEGF expression in myeloid cells resulted in a marked increase in the level of pericyte coverage, indicating vascular normalization and suggesting that VEGF expression from infiltrating myeloid cells is essential for intra-tumoral loss of vessel pericyte association. Interestingly, vessel permeability was also reduced in tumors from mutant animals, representing another indicator of vascular normalization. Consistent with the vascular changes and the concept of vascular normalization loss of myeloid-derived VEGF increased the efficacy of chemotherapeutic treatment (49).

Further studies suggested that hypoxia also upregulates the expression and secretion of ADM by macrophages (50), which are often regulated by HIF and VEGF (51, 52). A recent study showed that TAM-induced endothelial cell migration and tubule formation are inhibited by treatment with an ADM neutralizing antibody (53). These findings demonstrate that ADM can function as a novel pivotal factor of TAMs in facilitating tumor angiogenesis. TAMs also have the ability to release a number of other pro-angiogenic factors, including growth factors [such as PlGF, basic-fibroblast growth factor (b-FGF), M-CSF, PDGF, heparin-binding epidermal growth factor (HB-EGF), macrophage-inhibitory factor (MIF), platelet activating factor (PAF), and TGF-β], and cytokines (such as IL-1, IL-8, TNF-α, and MCP-1) (54, 55). Recent studies have increased our understanding about TAM-derived factors involved in angiogenesis. In solid tumors, the hypoxic condition often induces apoptosis of tumor cells (56). The apoptotic tumor cells can up-regulate prostaglandin E2 (PGE2) production from macrophages to promote angiogenesis (57). Semaphorin 4D (Sema4D) is a pro-angiogenic molecule that acts through its receptor, plexin B1 (58). In the tumor microenvironment, TAMs are the major source of Sema4D, which is critical for tumor angiogenesis and vessel maturation, as demonstrated by the impaired angiogenesis and vessel maturation in Sema4D knockout mice (59). In addition to producing pro-angiogenic factors in the hypoxic condition, TAMs also promote angiogenesis by downregulating the expression of angiogenesis inhibitors, such as vasohibin-2 (60).

Apart from the secretion of pro-angiogenic factors, TAMs also express a number of angiogenesis-modulating enzymes, such as COX-2, iNOS, and various matrix metalloproteinases (45, 61–64). For instance, TAM-derived MMP-9 is required for angiogenesis in a model of human cervical cancer (62). Cathepsin proteases are also implicated in human tumor progression (65). In the tumor microenvironment, TAMs represent an important source of cathepsins in pancreatic cancer and mammary tumor. Ablation of TAM-derived cathepsin B or S in these tumors impairs tumor angiogenesis, suggesting their critical roles in mediating TAMs effects on angiogenesis (66).

Recently, it has also been proposed that circulating monocytes transdifferentiate into endothelial cells and thereby contributing to tumor angiogenesis (67). However, whether recruited monocytes/macrophages significantly contribute to the formation of the tumor vasculature by this mechanisms remains to be further determined.

In summary, when TAMs are attracted to the hypoxic areas of tumor site, they produce a large body of pro-angiogenic factors in addition to angiogenesis-modulating enzymes, under the regulation of specific signaling pathways (i.e., NF-κB and mTOR) and transcription factors (i.e., HIFs and Stat3), which contribute to tumor angiogenesis. On the other hand, targeting angiogenic factors in TAMs may also promote tumor vessel normalization. A number of findings support the concept that TAMs are educated by tumor cells and tumor microenvironment, and “re-education” of TAMs is now emerging as a novel strategy for cancer therapies via tumor angiogenesis inhibition and vessel normalization.

### Neutrophil granulocytes

These phagocytes represent the largest population of blood leukocytes and are critical for the initial inflammatory reaction to invading microbes. Neutrophil infiltration has been reported in various cancer entities (68) and neutrophils are particularly abundant in the invasive front of the tumor (69, 70).

The CXC chemokine system plays a crucial role in neutrophil recruitment and transmigration via activation of the receptors CXCR1 and/or CXCR2 (68, 71–73). Particular attention has been paid to CXCL8 (IL-8), which is highly expressed in a large number of cancer types (74–76). Furthermore, CXCL8 expression in patients with bronchioloalveolar carcinoma correlates positively the number of tumor-associated neutrophils as well as with a poor prognosis for the patients (70). Likewise, in a mouse model of CXCL8-overexpressing (human) ovarian carcinoma, the tumors show increase infiltration of neutrophils (77). However, it is important to mention that there is redundancy within the CXC chemokine system. Hence, it is likely that a complex crosstalk between different CXC chemokines and the activation of their cognate CXC regulates the recruitment of neutrophil granulocytes to the tumor.

Various mouse models have shown that neutrophils are crucially involved in the process of tumor angiogenesis. Antibody-mediated depletion of neutrophils impaired angiogenesis in mice inoculated with CXCL8-containing matrigel plugs (78) as well as in the transgenic RIPK1-TAG2 mouse model of pancreatic carcinoma (79). Furthermore, tumor-infiltrating neutrophils are express high levels MMP-9 (80), and therefore could foster angiogenesis by releasing angiogenic factors from the extracellular matrix (81).

In addition, neutrophils are able to release angiogenic molecules like VEGF upon activation to induce vascular remodeling. However, whereas hypoxia induces the upregulation of VEGF expression in TAMs, neutrophil VEGF-release remains unaffected by the oxygen levels (82). In contrast, exposure to TNFα triggers the release of VEGF directly from neutrophils (83) and furthermore, TNFα induces the production of the angiogenic chemokines CXCL8 and CXCL1 (84, 85). Finally, CXCL8 can trigger neutrophil MMP-9 release, which in turn generates a highly active form of CXCL8 by means of protein cleavage (86), thereby creating feed forward loop involving release of angiogenic cytokines and additional neutrophil recruitment.

### Mast cells

Infiltrates of mast cells have been observed in solid tumors as well as hematological malignancies (87). Mast cells are able to release an array of angiogenic factors, including fibroblast growth factor (FGF)-2 and VEGF (88, 89). In experimentally induced tumors, mast cell infiltration precedes the angiogenic switch and the development of carcinomas from dysplastic cells (90–92).

Mast cell recruitment depends on the secretion of tumor-cell-derived soluble factors of which the stem cell factor (SCF) is considered to be the most important (93, 94). In addition FGF-2, VEGF, platelet-derived endothelial cell growth factor (PD-ECGF), RANTES, monocyte chemotactic protein (MCP)-1, adenosine, and adrenomedullin have been reported to play a role in mast cell trafficking (95–97).

In the transgenic mouse model human papilloma virus (HPV) 16-induced carcinogenesis showed mast cell accumulation around hyperplastic and dysplastic cells that preceded the onset of angiogenesis and malignant transformation (98). Infiltrating mast cells were indentified as important sources of MMP-9, tryptase, and chymase and noteworthy, in this setting angiogenesis was abrogated by mast cell-deficiency, highlighting the importance of mast cell-driven angiogenesis in squamous cell carcinogenesis. (98).

Likewise, in colon carcinomas that develop from premalignant polyps, the adenomateous polyps are characterized by high numbers of mast cells. Remarkably, existing polyps show significant remission upon mast cell depletion (99). Furthermore, the presence of mast cell and particularly their ability to degranulate has been shown to be indispensable for tumor progression in a Myc-driven model of pancreatic cancer. In contrast, preventing the degranulation of mast cells within the tumor stroma leads to rapid apoptosis of tumor cells as well as vascular endothelial cells (100).

Several experiments have shown that the expression of another important angiogenic factor, angiopoietin-1 (Ang-1) by mast cells drives neoangiogenesis (101). A close correlation between the presence of mast cells, neovascularization, and tumor progression has been shown for various tumor entities, including plasmacytoma, in mammary carcinoma (102, 103), colon cancer (104), and cervical cancer (105). In the latter tumor-associated mast cells are tryptase-positive and their number increases number along with vascular density during the transition from cervical dysplasia to invasive carcinoma of the cervix (106). Furthermore, accumulation of VEGF-expressing mast cells has been documented in laryngeal, pulmonary neoplasms, and malignant melanoma (107–114) and in the latter one this correlated with a poor prognosis (115). In esophageal and endometrial cancer as well as in hematological malignancies including B-cell non-Hodgkin’s lymphomas, multiple myeloma, myelodysplastic syndrome, and B-cell chronic lymphocytic leukemia, the degree of mast cell infiltration and vascular density have been shown to be of prognostic value (116–122).

### Eosinophil granulocytes

Eosinophils are specialized in defending the body against parasites by releasing granules loaded with highly cationic proteins and play a crucial role during allergies (123).

Accumulation of eosinophils has been documented in various tumor types including nasopharyngeal (124) and oral squamous cell carcinomas (125), tumors of the gastrointestinal tract (104), and lymphomas (126). However, whether the presence of eosinophils represents a positive or negative prognostic factor is tumor entity-dependent. Recruitment of eosinophils to the tumor depends on the chemokine CCL11, which is highly selective for this cell type (127, 128). Depending on the tumor context, tumor cells, fibroblasts, and endothelial cells in the tumor stroma as well eosinophils themselves have been identified as important sources for CCL11 within the tumor.

With regard to their angiogenic function, it has been suggested that eosinophil-stimulated proliferation and migration of endothelial cells is at least partially mediated by VEGF (129). Indeed, *in vitro*-cultured eosinophils release VEGF with their granules and the secretion of these granules is triggered by IL-5 (130, 131). However, whether this takes place to the same extent in the tumor microenvironment awaits further experimental evidence.

In addition to VEGF, eosinophil granules contain a diverse array of molecules that promote angiogenesis, including b-FGF, IL6, CXCL8, GM-CSF, PDGF, TGFβ (132), and MMP-9 (133). These angiogenic responses and the release of these molecules occur upon stimulation with TNF-α and CCL11 (133, 134). Interestingly, eosinophils preferentially infiltrate into hypoxic areas of the tumor (135). Therefore, degranulation of eosinophils and secretion of angiogenic factors in the tumor microenvironment might deliver the angiogenic signal specifically the hypoxic regions of the tumor.

### Myeloid-derived suppressor cells

Myeloid-derived suppressor cells (MDSC) represent a subset of immature progenitor cells for myeloid cells (136). MDSC can be roughly divided into CD11b+Gr1hi (alternatively LY6G+LY6Chi), which are reminiscent of immature neutrophils, and those that are CD11b+Gr1low (LY6G+LY6Clow) and present a monocyte-like phenotype (137, 138). MDSCs have a strong immunosuppressive function (137, 139, 140) and potently inhibit T and NK cell activity as well as DC maturation (141, 142).

Myeloid-derived suppressor cells can be detected in tumors as well as in the circulation of cancer patients and their numbers correlate with cancer stage (142–144). Noteworthy, therapy with cytotoxic agents can further increase the burden of circulating MDSCs, indicating that this cell type might play a role in treatment failure (143). Likewise, MDSCs can be found in various murine tumor models (140, 145–147) where they represent up to 5% of the cells (136).

Upon stimulation with G-CSF, CD11b+Gr1+ cells can directly contribute to vessel neoformation by releasing the protein Bv8 (146) with and its interaction via its receptors EG-VEGRF/PKR-1 and EG-VEGFR/PKR-2 (148). In addition, Bv8 can stimulate the mobilization of granulocytes and monocytes (149). Neutralization of Bv8 results in reduced vessel density and impaired tumor growth in xenograft tumor models as well as in transgenic model of pancreatic cancer (146, 150). Besides the suppression of the anti-tumor activity of T and NK cells by means of arginase 1 and inducible nitric oxide synthase (iNoS) (151–153), MDSCs can foster tumor growth by releasing MMPs that increase the bioavailability of VEGF within the tumor microenvironment (136, 147). Particularly, MMP-9 seems to play an important role in tumor vascularization since vessel formation and tumor growth was impaired in the presence of MMP-9-deficient MDSCs (136).

Interestingly, some MDSC populations are found in juxtaposition to tumor blood vessels, indicating that MDSC are actively retained in the perivascular area whereas other MDSCs seem to transdifferentiate into endothelial cell-like cells including increased expression of CD31 and VEGFR2 (136) and integrate into tumor vasculature. Hence, it will be key to identify the mechanisms that guide MDSC positioning within the tumor as well as the signaling pathways that regulate MDSC transdifferentiation.

### TIE2-expressing monocytes

A unique feature of the recently discovered TIE2-expressing monocytes (TEM) in contrast to other monocyte populations is that they express the angiopoietin receptor TIE2 (154–157). However, TEM are different from TIE2-expressing circulating endothelial cells or endothelial progenitors cells (157). The presence of TEM has been described in various human tumor entities (157) as well as in different mouse models of cancer (154). TEM recruitment is largely regulated by the TIE2 ligand, angiopoietin-2 (ANGPT2) (155, 157). Tumor-infiltrating TEM have been shown to localize in close proximity to blood vessels and to hypoxic areas of the tumor (154, 157). However, it is not known whether this distribution pattern is due to differential ANGPT2 expression (158, 159) within the tumor.

The localization of TEM adjacent to tumor blood vessels indicated that these cells might have profound impact on the process of tumor angiogenesis. Indeed, selective ablation of TEM from the tumor microenvironment reduced angiogenesis and impaired growth in gliomas (154) without affecting the recruitment of TAM or neutrophils into these tumors. Remarkably, despite the fact that TEM numbers within the tumor are lower than those of TAM and granulocytes, TEM showed significant contribution to vessel neoformation, further indicating that this cell type is a potent driver of tumor angiogenesis (154). Recently, it has been shown that TEM transmit the angiogenic signal at least partially by the expression of b-FGF (154). However, the mechanisms by which TEM exert they pro-angiogenic function are still matter of debate and subject of current studies.

### Natural killer cells

Natural killer cells are cells of the innate immunity that arise from a common lymphoid progenitor cell. These cells are characterized by a high cytolytic capacity against transformed cancer cells. In addition to their important role in immunosurveillance, NK cells can contribute to neovascularization, particularly in the uterus. In humans, uterine NK cells express high levels of CD56 and low levels of CD16 (CD56bright CD16dim) and can infiltrate the uterus in large numbers. These uterine NK cells show a highly angiogenic phenotype and contribute to the physiological vascular remodeling in the uterus during the secretory phase of menstrual cycle as well as during pregnancy (160).

However, the contribution of NK cells to the process of tumor angiogenesis has not been thoroughly dissected yet. A recent study showed that the CD56(+)CD16(−) NK subset in non-small cell lung cancer patients, which represents the predominant NK subset in tumors, was associated with VEGF, placental growth factor (PIGF), and interleukin-8 (IL-8)/CXCL8 production. Peripheral blood CD56(+)CD16(−) NK cells from patients with the squamous cell carcinoma subtype showed higher VEGF and PlGF production compared to those from patients with adenocarcinoma and controls. This suggests that NK cells in non-small cell lung cancer act as pro-angiogenic cells (161).

Furthermore, a recent study identified (NCR) NKp46-expressing lymphoid tissue inducer cells to play an important role in IL-12 mediated tumor rejection. Interestingly, tumor rejection by these cells did neither depend a cytokine response involving IFN-γ, IL-22, lymphotoxin, or IL-17 nor perforin-dependent cytotoxic activity. Instead, NKp46+ lymphoid tissue inducer cells induced the expression of various adhesion receptors by tumor endothelium thereby facilitating the infiltration of other pro-angiogenic leukocytes into the tumor (162).

Yet, the precise role for NK cells in tumor angiogenesis remains to be defined. Given the crucial impact of NK cells on the phenotype of the uterine vasculature, it will be important to define the role of NK cells for vascular remodeling of the tumor vasclature by combining NK cell-specific deletions of angiogenic factors with murine models of cancer.

### Dendritic cells

Dendritic cells play a pivotal role in tuning the adaptive immune response owing to their highly specialized function of antigen presentation and the ability to trigger both primary T- and B-cell responses. DC can be roughly divided into two subpopulations: myeloid DC (MDC) and plasmacytoid DC (PDC) (163, 164).

Myeloid DC in the bone marrow are immature dendritic cells with a high phagocytic potential. The maturation of these cells is usually initiated upon antigen processing, which also leads to homing of DC to secondary lymphoid tissues. In a subsequent step DC can trigger the activation of antigen-specific T cells. Interestingly, recent studies could show that soluble factors derived from the tumor can interfere with this maturation process and impair the development of mature DC (165, 166). Consistent with this, tumors frequently exhibit an accumulation of immature DC and only very few mature MDC (167, 168). Among the tumor-derived factors that potentially recruit immature DC to the tumor, VEGF (165, 169), β-defensin (170), CXCL12 (171), HGF (172), and CXCL8 (173) have been suggested.

Tumor-associated DC can directly drive tumor angiogenesis through the release of pro-angiogenic cytokines such as TNFα, CXCL8, and osteopontin (171, 173). Moreover, these factors stimulate other cells including monocytes to release pro-angiogenic molecules such as IL-1 (174–176). Furthermore, recent work indicates that immature DC which coexpress DC and endothelial markers represent a reservoir of endothelial progenitor cells. After transdifferentiation into endothelial-like cells, these cells are able to integrate into the vasculature and thereby foster tumor angiogenesis (177). Interestingly, the expression of endothelial cell markers in DC and the process of transdifferentiation seem to be controlled by the angiogenic factors VEGF and oncostatin M (170, 178).

Among the tumor-derived factors that might be responsible for the angiogenic phenotype of immature DC, VEGF has been most extensively studied (169, 179). However, other tumor-derived factors like HGF (172), TGFβ (180), prostaglandin E2 (181), lactate (182), and osteopontin (183) are also involved in the suppression of DC maturation and the induction of pro-angiogenic properties. Conversely, immature DC can increase the expression of VEGF and CXCL8 upon hypoxic challenge (184), which might exert pro-angiogenic function in the tumor microenvironment (169, 173, 185).

## Adaptive Immunity

### B cells

The impact of B cells on inflammation-associated cancer development remains to be further explored. High numbers of B lymphocytes have been found in aggregates with other immune cells at the inflammatory site in tumor tissues of various human cancers (186). The intra-tumoral presence of B cells together with CD8^+^ T cells has been correlated with enhanced survival in patients of ovarian (187) and non-small lung cancer (188) in contrast to tumor tissue with exclusively one cell population, and beneficial effects of B-cell-mediated antibody production have been shown to result in better prognosis for patients of medullary breast cancer (189). Beside the beneficial effect of B cells on anti-cancer immunity, mechanistic *in vivo* studies also identified a cancer-promoting role of this cell type. Increased immunoglobulin deposition, triggered by B cells, promote enhanced recruitment of immune cells into premalignant skin and, in consequence, resulted in malignant progression during chronic inflammation in experimental skin cancer models (190, 191). Exemplarily, adoptive transfer of B cells into B- and T-cell deficient mice has been shown to restore the phenotype of activated tumor vasculature (190). Quite recently, Yang and colleagues identified the interplay between B cells with ECs via the signal transducer and activator of transcription 3 (STAT3) (192) an established and critical mediator of tumor angiogenesis caused by its potential to regulate VEGF expression (193–195). By using different experimental tumor models they could show that B cells differ in their function in dependence of STAT3 expression. STAT3 was persistently activated in tumor-infiltrating B cells during tumor growth. Adoptive transfer of intrinsic activated STAT3-expressing B lymphocytes into implanted Rag1^−/−^ mice, lacking mature T or B cells resulted, contributed to tumor growth and progression whereas in turn, adding STAT3-deficient B cells to the tumor microenvironment resulted in reduced tumor development. Furthermore, the impact of Stat3 activity in B cells for tumor progression was accompanied by enhanced tumor angiogenesis representing increased numbers of tumor-associated blood vessels (192). Further analyses identified the upregulation of several STAT3-downstream pro-angiogenic molecules such as VEGF in ECs after reciprocal interaction with STAT3-activated B cells (192). Nevertheless, B cells also have the potential to modulate tumor angiogenesis via interaction with myeloid cells. It is well known that in progressing tumors, TAMs generally represent the M2-like polarization, which is characterized by low inflammatory but high tissue remodeling and pro-angiogenic potential (34, 196). The polarization of TAMs is orchestrated by tumor- and host-derived cytokines and chemokines (6). In the majority of human cancers high amounts of TAMs in tumor infiltrates correlates with bad prognosis and reduced overall survival (197, 198). A recent study using a HPV-driven mouse model of squamous cell carcinoma indicates that B-cell-produced antibodies have a key role in macrophage-driven tumor progression by interaction and activation of Fcγ receptors on both tumor-resident and recruited myeloid cells. As a result, immune complex-triggered TAMs recruit myofibroblasts via macrophage-derived IL-1 into the tumor site, which in consequence, promote tumor angiogenesis (199). These studies pointed out the significance of B-cell-mediated pathways for therapeutic intervention in patients with chronic inflammatory disease. Initial clinical trials targeting B cells and IL-1 are currently running (200, 201) and may provide more insights into defining the diversity of cancer-related inflammatory response in humans, as well as may offer new innovative anti-tumor strategies.

### T cells

Circulating T lymphocytes interact with human vascular ECs that express class I and II MHC-peptide complexes but also a variety of different co-stimulatory molecules on their surface by attachment and transmigration through capillaries (202). So, whenever foreign peptides, such as microbial pathogens, are presented by endothelial MHC molecules, this contact-dependent interaction offers the opportunity to trigger circulating T-cell response. Undeniable, the MHC expression patterns vary among species and tissue. Exemplarily, the expression level of MHC molecules in non-lymphoid tissues has been found to be much higher than on other cells (203) and also class II MHC molecules have been detected on ECs throughout the human microvasculature and veins but its expression varies on arteries dependent on anatomic location (204). However, T cells can directly regulate the level of MHC expression via IFN-γ secretion (205, 206) but also influence the regulatory function of ECs namely the regulation of blood vessel formation and remodeling, blood flow, permselectivity, blood fluidity, and hemostasis (207). Although T cells are not a source of classical angiogenic modulators such as VEGF or angiopoietin-2, they can directly synthesize b-FGF and heparin-binding epidermal-like growth factor (HB-EGF), acting in a pro-angiogenic manner (208). On the other hand, also inhibitory properties of T-cell-derived cytokines such as TNF, TGF-β, and INF-γ on angiogenic processes have been reported *in vitro* and *in vivo* (209–212). Paradoxically, TNF can also act in a pro-angiogenic manner through induction of sphingosine-1-phosphate, which in turn interacts with Edg family receptors on ECs (213). Via surface-bound molecules like TNF, FasL, or Trail, T cells can also induce cell-contact-dependent apoptosis of ECs (214). In accordance with TNF-induced apoptosis, killing via FasL appears to require sensitization of the endothelium, e.g., by IFN-γ-induced upregulation of Fas and pro-caspase-8 (214).

T-cell-secreted TNF can also influence the blood fluidity by converting EC from an anti-thrombotic to a pro-thrombotic state via production of pro-coagulant proteins such as tissue factor (TF) and plasminogen activator inhibitor-1 (215). In parallel, TNF has the potential to diminish thrombomodulin expression by transcription inhibition (216). The physiological synergy between activating responses, such as the induction of TF expression, and dysfunctional responses like the loss of thrombomodulin which, in consequence, up-regulates fibrin deposition on the surface of vascular ECs may underlie pathophysiological processes such as intravascular thrombosis in a variety of vascular diseases.

T-cell-derived cytokines also allow T cells to influence the cytoskeletal rearrangement in EC by stimulation of gap junction formation, resulting in reduced vascular permselectivity of cultured ECs (217), but also cytokine-independent T-cell contact-dependent vascular leakage has been reported (218) of which the mechanism behind is already unknown. Heterotypic gap junctions between T cells and ECs have also been reported during the course of autoimmune inflammation (219).

Importantly, T-cell-derived TNF, IL-1, and INF-γ have been implicated in the regulation of the inhibitory molecule programed cell death-1 ligand (PD-L1) (220). This is of high importance since a successful anti-tumor immunotherapy requires not only activated tumor antigen-specific T cells, but also the access of T-cell to the malignant compartment by a vascular network. Recent studies suggest that lower dosage of anti-angiogenic treatment may be pave the way forward a “normalized” tumor vessel situation, which, in turns, result in a more effective strategy to recondition the tumor immune microenvironment for anti-cancer immunotherapies in a clinical setting such as blockage of immune checkpoints. For example, the ongoing clinical trial using an anti-PD-L1 antibody in combination with a high dose of bevacizumab (anti-VEGF antibody) in patients with advanced solid tumors might shed some light on this interaction (see ClinicalTrails.gov, NCT01633970).

## Conclusion

**Figure 1:**
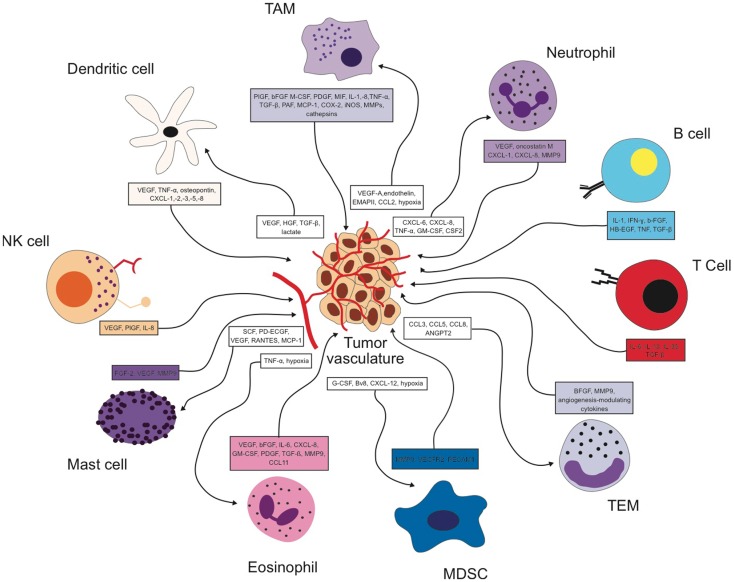
**Reciprocal interactions between different immune cell types and the tumor vasculature in the tumor microenvironment**.

It is now recognized that the immune cell compartment within the tumor is a major driver of angiogenesis and vascular remodeling in addition to the tumor cell itself. As summarized in Figure 1, every immune cell type identified so far has been shown to impact the process of tumor angiogenesis either directly or indirectly. Furthermore, many angiogenic signaling pathways like VEGF are shared by the different cell types (Figure 1) so that targeting the angiogenic signal in one cell type could be compensated by another cell type. Similarly, the angiogenic signal can be transmitted by different factors. Therefore, inhibiting one factor might lead to the compensatory upregulation of another angiogenic molecule resulting in a rather modest effect on net angiogenic activity. Hence, overall angiogenic activity within a tumor is influenced by many different immune cell types and an even larger repertoire of angiogenic factors. It will be the future challenge to dissect out and understand how the interplay between all these different sources of pro-angiogenic stimuli is orchestrated. On the other hand, delivery of some angiogenic factors by certain immune cell subsets seems to play a strictly non-redundant role at least in a context-dependent manner. It is therefore of utmost importance to identify the players that provide exclusive angiogenic signals to the tumor microenvironment.

## Conflict of Interest Statement

The authors declare that the research was conducted in the absence of any commercial or financial relationships that could be construed as a potential conflict of interest.
